# Seaweed, Used as a Water-Retaining Agent, Improved the Water Distribution and Myofibrillar Protein Properties of Plant-Based Yak Meat Burgers Before and After Freeze–Thaw Cycles

**DOI:** 10.3390/foods14142541

**Published:** 2025-07-21

**Authors:** Yujiao Wang, Xinyi Chang, Yingzhen Wang, Jiahao Xie, Ge Han, Hang Qi

**Affiliations:** National Engineering Research Centre for Seafood, State Key Laboratory of Marine Food Processing and Safety Control, College of Food Science and Technology, Dalian Polytechnic University, Dalian 116034, China; wangyj202012@163.com (Y.W.); changxinyi1030@163.com (X.C.); wangyz2036@163.com (Y.W.); 19551597888@163.com (J.X.)

**Keywords:** yak meat, recombinant food, product development, seaweeds, frozen

## Abstract

This study investigated quality changes in seaweed–yak patties before and after freeze–thaw by varying seaweed addition levels (10–70%). Macroscopically, the effects on water-holding capacity, textural properties, and oxidative indices of restructured yak patties were evaluated. Microscopically, the impact of seaweed-derived bioactive ingredients on patty microstructure and myofibrillar protein characteristics was examined. LF-NMR and MRI showed that 40% seaweed addition most effectively restricted water migration, reduced thawing loss, and preserved immobilized water content. Texture profile analysis (TPA) revealed that moderate seaweed levels (30–40%) enhanced springiness and minimized post-thaw hardness increases. SEM confirmed that algal polysaccharides formed a denser protective network around the muscle fibers. Lipid oxidation (MDA), free-radical measurements, and non-targeted metabolomics revealed a significant reduction in oxidative damage at 40% seaweed addition, correlating with increased total phenolic content. Protein analyses (particle size, zeta potential, hydrophobicity, and SDS-PAGE) demonstrated a cryoprotective effect of seaweed on myofibrillar proteins, reducing aggregation and denaturation. These findings suggest that approximately 40% seaweed addition can improve the physicochemical stability and antioxidant capacity of frozen seaweed–yak meat products. This work thus identifies the optimal seaweed addition level for enhancing freeze–thaw stability and functional quality, offering practical guidance for the development of healthier, high-value restructured meat products.

## 1. Introduction

Meat products have long been the focus of nutritional and consumer preference studies because certain components (e.g., saturated fats and cholesterol) are closely associated with chronic diseases such as cancer, hyperlipidemia, and obesity [1,2]. Consequently, functional meat-based foods are being developed to deliver comprehensive nutrition and better satisfy consumer demand. Incorporating meat with ingredients rich in bioactive compounds can significantly enhance the intake of health-promoting substances [2]. Data show that 55.9% of consumers prefer burger-style convenience foods [3]; moreover, among these, restructured beef steaks offer both ease of consumption and balanced nutrition [4]. Since dietary fiber content in meat is relatively low, adding fiber-rich materials can help mitigate the risk of certain diseases [4,5]. Seaweeds are abundant in natural phytochemicals, including dietary fibers, polysaccharides, and phenolics, [3]. and studies have demonstrated that integrating these bioactive substances into meat products not only reduces health risks associated with processed meats, but also improves the quality of restructured foods [3,6]. For example, the addition of 0.5% brown-algae extract (rich in algal polysaccharides and alginates) to pork patties markedly decreased lipid oxidation in cooked products while enhancing antioxidant capacity and textural properties [7]. He et al. found that incorporating 0.4% Eucheuma spinosum into chicken-breast surimi improved water-holding capacity (WHC) [8], and edible seaweeds added to frankfurters significantly enhanced texture, sensory attributes, and oxidative stability [9]. Freezing at −18 °C is a core preservation method for meat products, extending shelf life and inhibiting microbial growth; however, freeze–thaw cycles can damage muscle fibers, alter texture and color, and accelerate protein and lipid oxidation [10,11]. During the freezing process, the distribution of water molecules within the cells changes, leading to the formation of ice crystals, which damages the muscle fibers, affects the texture of the food, alters its color, and accelerates the oxidation of proteins and fats upon thawing [12]. Furthermore, during the thawing process of frozen meat products, the destruction of muscle fiber structure prevents the muscle cells from reabsorbing the externally migrated water, resulting in the loss of juice in pre-cooked steaks [11]. These changes adversely affect the quality of prepared foods; therefore, it is essential to prevent moisture loss caused by ice crystal growth during the freezing and thawing processes while ensuring the nutritional balance of prepared foods. Reports indicate that cryoprotectants, such as alginate [13], ice structuring proteins [14], and certain plant extracts [15], can enhance the functional quality of prepared foods. In frozen meat products, a compound of 4% sucrose and 4% sorbitol can serve as an effective cryoprotectant [16]. These agents interact with proteins through non-covalent forces such as hydrogen bonding, electrostatic interactions, or hydrophobic effects, replacing the water molecules surrounding the protein. This mechanism significantly inhibits protein denaturation caused by the freezing process [17]. For instance, after freezing at −18 °C, fish surimi products containing 2.77 g kg^−1^ edible Ulva intestinalis powder exhibited significantly improved oxidative stability, pH, color, texture, cooking yield, and sensory scores compared to a control group [18]. Likewise, the incorporation of brown-algal polyphenols and α-tocopherol effectively suppressed oxidation during 90 days of −18 °C storage, preserving gel structure in fish surimi [19]. In this study, yak beef was selected due to its pollutant-free rearing at a high altitude, unique flavor, tender texture, and rich nutrient profile [20]. Although the yak meat industry has great potential to meet growing demand, low-value cuts remain underutilized, and the meat’s inherent coarseness and coarse muscle fibers present challenges. Furthermore, few studies have investigated how algae addition affects the quality of restructured, plant-based yak beef products before and after freeze–thaw cycles. Therefore, this work aims to evaluate the effects of varying seaweed inclusion levels on water-holding capacity, textural properties, oxidative stability, microstructure, and myofibrillar protein characteristics of restructured yak beef patties during freeze–thaw cycles in order to identify the optimal formulation for enhancing functional quality and stability. This research not only offers a novel approach for valorizing low-value meat cuts, but also provides fresh insights into mitigating quality degradation in restructured, plant-based yak beef products during freezing and thawing.

## 2. Materials and Methods

### 2.1. Sample Preparation

#### 2.1.1. Pretreatment of Seaweed

*Laminaria japonica* (*L. japonica*) was purchased from Metro Supermarket (Dalian, China). *L. japonica* was immersed in deionized water and washed three to four times with deionized water. It was then immersed in water for 2 h until it reached a swollen state. Excess surface moisture was gently blotted away with filter paper, and the kelp was cut into 6 cm × 3 cm pieces. These pieces were ground in a laboratory mill, then stored in sealed, food-grade containers at 4 °C and used within 24 h.

#### 2.1.2. Collection of Yak Meat Samples

In this experiment, nine healthy, disease-free male yaks (mean age 3 years; body weight 400 ± 20 kg) were randomly selected from Gannan Tibetan Autonomous Prefecture, Gansu Province, China. All animals were humanely slaughtered at a professional meat-processing facility in accordance with the People’s Republic of China national standard “Regulations for Cattle Slaughter Operations” (GB/T 19477-2018) [21]. Three batches of experiments were carried out. Immediately after slaughter, 10–12 kg of longissimus thoracis was excised from each carcass, trimmed of surface fat and fascia, vacuum-packaged, and aged overnight (approximately 12 h) at −18 °C. The samples were then transported under cold-chain conditions to the laboratory and stored at −20 °C in an ultra-low-temperature freezer for standardized frozen storage. To ensure sample homogeneity, each 3 kg batch of yak meat was prepared by randomly sampling the longissimus thoracis from three carcasses and blending them.

#### 2.1.3. Preparation of Seaweed–Yak Meat Patties

**Figure 1:**
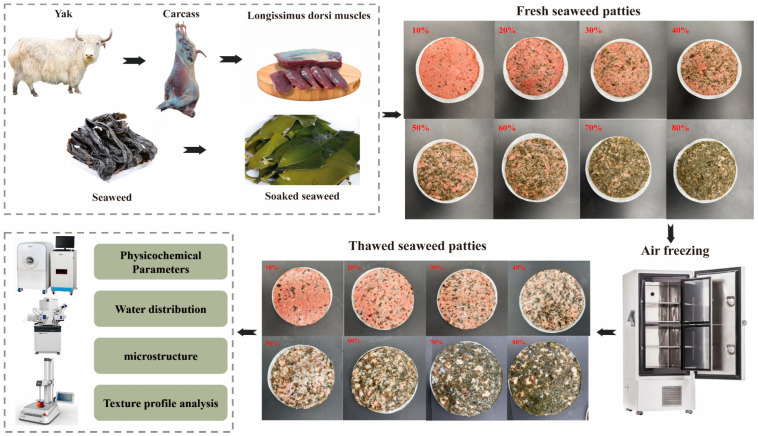
Experimental design schematic for assessing the quality characteristics of plant-based yak meat burgers before and after freeze-thaw.

In this experiment, eight yak meat patty formulations containing 10%, 20%, 30%, 40%, 50%, 60%, and 70% *L. japonica* were prepared. The experimental workflow is illustrated in Figure 1. Frozen yak meat samples (−20 °C) were first removed and placed on clean stainless-steel trays, then thawed in a temperature-controlled chamber (Model SCR-90; 20 ± 1 °C; 50 ± 5% RH; 0.3 m/s forced air) until the core temperature reached −5 °C to 0 °C, as monitored in real time with a digital probe. After thawing, the meat was cut into small pieces using knives sterilized at 121 °C and pre-chilled at 4 °C, then ground in a pre-cooled (4 °C) meat grinder. The ground seaweed and meat were combined in sterile mixing bowls at the eight ratios specified in Table 1 and stirred at 12 ± 1 °C until the *L. japonica* was uniformly dispersed. Patties (7 cm diameter × 2 cm thickness) were formed, each weighing approximately 50 ± 0.05 g. The fresh patties were analyzed immediately, and the remaining samples were stored overnight at −20 °C and thawed as described above prior to further testing.

### 2.2. Measurements of Water-Holding Capacity

#### 2.2.1. Thawing Loss

Thaw loss was determined by measuring the weight difference before and after freeze–thaw treatment. Initially, the fresh samples were weighed and labeled as M_0_. After the freeze–thaw cycle, the samples were blotted with filter paper to remove surface moisture, weighed again, and labeled as M_1_ [22]. Each group was measured in triplicate, and the formula is as follows:

$$Thawing\;loss\left(\%\right)={(M}_{0}-M_{1})/M_{0}\times 100$$

#### 2.2.2. Centrifugal Loss

The water-holding capacity of the sample was measured using a centrifugation method [23]. Initially, the sample was cut into cubes measuring 2 cm × 2 cm × 1.5 cm, weighed, and recorded as W_1_. Then, the sample was wrapped in filter paper and centrifuged at 5000× *g* for 10 min at 4 °C (Eppendorf AG, Hamburg, Germany). After centrifugation, the sample was weighed again and recorded as W_2_. Each group of samples was measured in triplicate, and the formula is as follows:

$$WHC\left(\%\right)={(W}_{1}-W_{2})/W_{1}\times 100$$

#### 2.2.3. Water Distribution and Migration

Low-field nuclear magnetic resonance (LF-NMR, MesoQMR23-060H, Shanghai Niumag Analytical Technology Co., Ltd. Shanghai, China) was used to measure water distribution and migration [24]. The sample was first cut into small cubes measuring 2 cm × 2 cm × 1.5 cm and placed on the sample tray for measurement. The Q-CPMG sequence was employed to measure the transverse relaxation time (*T*_2_) of water in the sample before and after freeze–thaw, with a waiting time (*TW*) of 5000 ms and an echo time (*TE*) of 0.2 ms. Additionally, images of the H-proton density were obtained using an NMR imaging system.

### 2.3. Determination of Physicochemical Characteristics

#### 2.3.1. Color Measurement

The L*, a*, and b* values of the samples were measured before and after freeze–thaw treatment using the UltraScan Pro colorimeter (HunterLab, Reston, VA, USA) [25]. In addition, the total color difference (Δ*E*) was calculated using the following formula:

$$∆E=\sqrt{{\left(L^{*}-L_{0}^{*}\right)}^{2}+{\left(a^{*}-a_{0}^{*}\right)}^{2}{+\left(b^{*}-b_{0}^{*}\right)}^{2}}$$

#### 2.3.2. pH Measurement

After preparing the samples before and after freeze–thaw treatment, the TESTO 205 portable pH meter (TESTO AG, Lenzkirch, Germany) was inserted into three different positions of each sample. During the measurement process, we ensured the probe was in full contact with the sample before recording the results [26].

#### 2.3.3. TPA and Shear Force Analysis

After cutting the samples, before and after freeze–thaw, into small cubes measuring 3 cm × 2 cm × 1.5 cm, the texture properties and shear force values were measured using the TA.XT.plus (Stable Micro Systems, Godalming, Surrey, UK). The P/50 probe with a test spacing of 20 mm, a deformation rate of 50%, and a speed of 5 mm/s was selected. Each sample was measured in parallel three times [27].

#### 2.3.4. Evaluation of Sensory Methodologies

The sensory evaluation of seaweed–yak-beef patties was conducted with reference to Ozogul et al. (2017) [28] with slight modifications. Fifteen professionally trained panelists (aged 23–30) with prior sensory assessment experience participated. No interaction or communication was permitted during scoring. Samples were maintained at a constant temperature (22 ± 1 °C) and presented in randomized order on white porcelain dishes under standardized lighting. Odor: The panelists brought the sample to within 5 cm of the nose and inhaled gently 2–3 times before assigning an odor score. Color: Each panelist viewed the sample perpendicularly from a distance of 20 cm and scored the surface and internal color. Texture and Elasticity: The panelists manipulated the sample between the thumb and forefinger to assess the surface texture, then took a single bite to evaluate mouthfeel and elasticity, rating chew resistance and spring back. Detailed scoring scales and attribute definitions are provided in Table 2.

### 2.4. Scanning Electron Microscopy

The microstructure of the samples was examined by scanning electron microscopy (SEM; JSM-IT500HR, JEOL Ltd., Tokyo, Japan), based on Zhao et al. (2020) [29] with minor modifications. The samples were trimmed into 2 × 2 × 1.5 cm cubes and fixed in 2.5% glutaraldehyde for 2 h, then rinsed twice with phosphate-buffered saline (0.1 M, pH 7.2). Dehydration was performed through an ethanol series (30%, 50%, 70%, and 100%), followed by immersion in an ethanol–tert-butanol mixture (1:1) and in pure tert-butanol for 15 min each. After treatment, the specimens were freeze-dried and sputter-coated with gold using an SCD 040 sputter coater (Bal-Tec AG, Pfäffikon, Switzerland) for 2 min. SEM observations were conducted at an accelerating voltage of 10 kV.

### 2.5. The Effect of Seaweed on the Oxidation Reaction

#### 2.5.1. Total Phenolic Content

The total phenolic content (TPC) was determined using the Folin–Ciocalteu method [2]. A 100 µL aliquot of the original sample (extracted at a concentration of 1000 µg/mL in water) was mixed with 2.0 mL of 2% Na_2_CO_3_ and left to stand at room temperature for 2 min. Subsequently, 100 µL of 50% Folin–Ciocalteu phenol reagent was added. After incubating in the dark at room temperature for 30 min, absorbance was measured at 720 nm using a spectrophotometer (Milton Roy Spectronic 1201, Milton Roy Co., Rochester, NY, USA). The total phenolic content was expressed as mg gallic acid equivalents (mg GAE/100 g fresh weight, fw).

#### 2.5.2. TBARS

Referring to the method of Zhao et al. (2020) [29], 2 g of the samples was taken in a test tube, and 1% thiobarbituric acid 3 mL and 2.5% trichloroacetic acid 17 mL were added sequentially, mixed, and then boiled in a boiling water bath for 30 min. Then, the cooled reaction samples were mixed thoroughly with trichloromethane 1:1 and centrifuged at 3000 r/min for 10 min. The absorbance values were measured at 531 nm. The TBARS value is calculated using the following formula:

$$TBARS(\mathrm{mgMDA/kg})=\frac{A_{523}}{M_{s}}\times 9.48$$

In the formula, A531 denotes the absorbance of the sample at 531 nm; Ms denotes the mass of the sample; 9.48 denotes the dilution factor and molar extinction coefficient of the reaction product from thiobarbituric acid.

#### 2.5.3. DPPH Radical Scavenging Activity

The antioxidant activity of yak meat patties before and after freeze–thaw treatment was measured using an A200 electron spin resonance (ESR) spectrometer (Bruker, Karlsruhe, Germany), following the method described by Wang et al. (2023) [30]. Specifically, 10 g of sample was weighed into a conical flask, followed by the addition of 50 mL of 80% anhydrous ethanol. The mixture was stirred for 30 min and then centrifuged. The resulting supernatant was incubated with DPPH for 30 min, after which ESR spectral analysis was performed.

#### 2.5.4. Non-Targeted Metabolomics Analysis

The samples (200 µL each) were first frozen in liquid nitrogen, then disrupted using a low-temperature, nitrogen-assisted homogenizer at 5500 rpm for 20 s, which was repeated three times with intermittent pauses. Next, 800 µL of a methanol–acetonitrile mixture (1:4 *v*/*v*) was added, the samples were vortexed for 30 s, and they were subjected to ultrasonic extraction for 10 min. To precipitate proteins, the homogenate was incubated at room temperature (20 °C) for 60 min, then centrifuged at 13,000× *g* for 15 min at 4 °C. The supernatant was collected and vacuum-dried, then reconstituted in 100 µL of 50% acetonitrile in water. After 10 min of ultrasonic-assisted dissolution, the centrifugation step was repeated, and the final extracts were stored at −80 °C until analysis.

Chromatographic separation and mass spectrometric detection were performed on a combined system consisting of a TripleTOF 6600+ high-resolution mass spectrometer (AB Sciex Pte. Ltd.; Woodlands Central Industrial Estate, Singapore) coupled to an Agilent 1290 Infinity II UPLC. Separation was achieved on a Waters ACQUITY UPLC BEH C18 reversed-phase column (100 × 2.1 mm, 1.7 µm) at 40 °C, with a flow rate of 0.5 mL/min, an injection volume of 5 µL, and a 12-minute gradient elution. Mass spectrometry was run in polarity-switching mode with an electrospray ionization (ESI) source using information-dependent acquisition (IDA) over an *m*/*z* range of 60–1200. The ionization parameters were as follows: spray voltage 5.0 kV (positive mode) and 4.0 kV (negative mode), source temperature 600 °C, curtain gas 35 psi, nebulizer gas 60 psi, auxiliary gas 60 psi, and collision energy 30 eV [31].

### 2.6. Changes in Protein Characteristics Before and After Freezing

#### 2.6.1. Extraction of Myofibrillar Protein

The yak meat samples were thawed at 4 °C for 12 h and then chopped. A 4 g sample was accurately weighed and mixed with 10 times its volume (*v*/*w*) of extraction buffer (containing 2 mmol/L MgCl_2_, 20 mmol/L potassium phosphate buffer, 1 mmol/L EGTA, 0.1 mol/L KCl, pH 6.8). The mixture was centrifuged at 2000× *g* for 10 min at 4 °C using a GL-20G-II centrifuge (Anting Scientific Instrument Factory, Shanghai, China). After discarding the supernatant, the centrifugation step was repeated twice. The pellet was then resuspended in 8 times its volume (*v*/*w*) of 0.1 mol/L KCl solution and centrifuged again at 2000× *g* for 10 min at 4 °C. After discarding the supernatant, this step was repeated twice more. Finally, the protein concentration of the obtained sample was determined using the biuret method, with bovine serum albumin (BSA) as the standard.

#### 2.6.2. Protein Particle Size and Zeta Potential

The particle size distribution was determined using an NS-90Z laser diffraction particle size analyzer (Zhuhai Omik Instrument Co., Ltd., Zhuhai, Guangdong Province, China), following the method established by Zhang et al. (2020) [32]. The samples were prepared as a 10 mg/mL protein solution for testing. The instrument parameters were set as follows: the sample refractive index was set to 1.520, and the dispersing medium was water with a refractive index of 1.333.

The zeta potential of the MP-KC mixed sols was measured using an NS-90Z potentiometer, following the method described by Li et al., with minor modifications (Zhuhai Omec Instrument Co., Ltd., Zhuhai, Guangdong Province, China) [33].

#### 2.6.3. Surface Hydrophobicity

A 5 mg/mL solution was mixed with a 1 mg/mL BPB solution, vortexed, and then centrifuged at 8000 rpm for 15 min. The supernatant was diluted 10 times with PBS (0.1 M NaCl, pH 7.0), and absorbance was measured at 595 nm [34].

$$BPB\left(µg\right)=200µg\times \left(S_{control}-S_{sample}\right)/S_{control}$$

#### 2.6.4. Sodium Dodecyl Sulfate-Polyacrylamide Gel Electrophoresis (SDS-PAGE)

A 1 mg/mL MP solution (in 20 mM Tris-HCl buffer containing 0.6 M KCl) was mixed with 5% SDS, centrifuged, and the supernatant was combined with sample buffer (containing 0.5 M Tris, 4% SDS, 20% glycerol, 10% β-mercaptoethanol, pH 6.8) in a 1:1 ratio, followed by heating in a boiling water bath for 5 min. Electrophoresis was carried out using 5% stacking gel and 12% separating gel at a constant voltage of 80 V until the bromophenol blue marker reached the bottom of the gel. The gel was stained with Coomassie Brilliant Blue for 30 min, destained in acetic-acid–methanol solution for 12 h, and then imaged [33].

### 2.7. Statistical Analysis

All experiments were conducted in triplicate, each using freshly prepared myofibrillar protein (MP) samples. One-way analysis of variance (ANOVA) was performed using IBM SPSS Statistics (Version 30.0; IBM Corp., Armonk, NY, USA) with a significance level of 0.05. Data are presented as mean ± standard error, and Duncan’s multiple-range test was used for multiple comparisons among the treatments. Heatmaps were generated and analyzed in R (version 3.6.1; R Foundation for Statistical Computing, Vienna, Austria). Graphs were plotted in Origin 2021, and all figures and data visualizations were compiled in PowerPoint 2019.

## 3. Results and Discussion

### 3.1. The Effect on Water-Holding Capacity

#### 3.1.1. Thawing Loss and Centrifugal Loss

**Figure 2:**
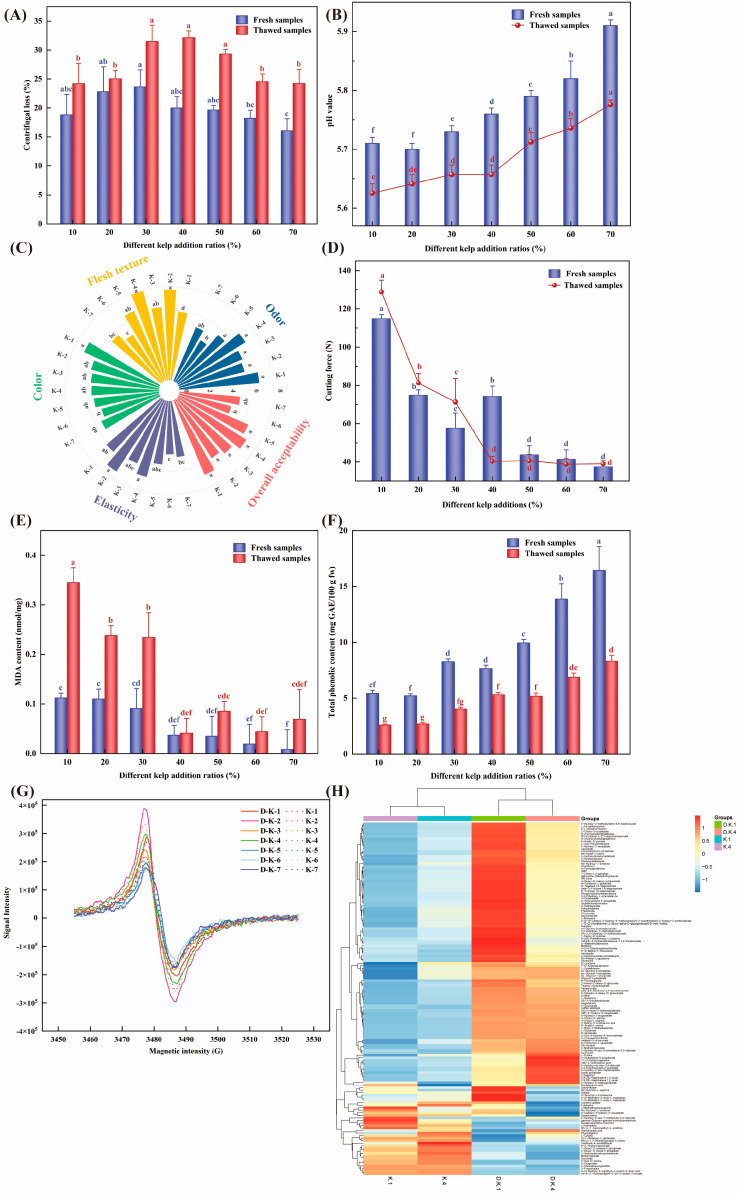
(**A**,**B**) represent the changes in centrifugation loss and pH value, respectively, of seaweed–yak patties with different seaweed addition levels before and after freeze–thaw treatment. (**C**) illustrates the effect of varying seaweed addition levels on the sensory characteristics of the patties. (**D**) indicates the change in shear force before and after freeze–thaw treatment. (**E**–**G**) correspond to the changes in MDA content, total phenolic content, and DPPH scavenging activity, respectively, under different seaweed addition levels before and after freeze–thaw treatment. (**H**) represents the hierarchical cluster analysis of seaweed–yak patties with 10% and 40% seaweed addition before and after freeze–thaw treatment. Lowercase letters in the figure denote differences among treatments at varying seaweed addition levels in both the fresh and freeze–thaw groups (*p* < 0.05).

Water loss during thawing affects the water-holding capacity of meat and meat products, altering weight, sensory quality, and nutrient content [35]. This study elucidated the effects of adding varying proportions of seaweed on thawing loss and centrifugation loss in restructured yak steaks. As shown in Table 3, thawing loss decreased progressively with increasing seaweed content, and the sample containing 40% seaweed exhibited significantly lower thawing loss than all other addition groups (*p* < 0.05), indicating that seaweed addition reduces thawing loss in restructured steaks. However, centrifugation loss showed an upward trend. Centrifugation loss is also a critical quality indicator for meat and meat products, directly affecting tenderness, juiciness, and weight changes during processing and storage [4,11]. Figure 2A illustrates that, compared with pre-freeze–thaw samples, thawed restructured steaks had higher centrifugation losses, with the 30%, 40%, and 50% seaweed-added groups exhibiting significantly greater loss than the other samples (*p* < 0.05). Notably, the 40%-seaweed group showed the most pronounced increase in centrifugation loss after thawing. Zhang et al. (2023) [36] also reported that adding composite dietary fibers to restructured meat products significantly increases centrifugation loss. The correlation between centrifugation and thawing loss trends suggests that seaweed addition functions as a cryoprotectant, effectively inhibiting water migration and enhancing structural stability, thereby improving meat product quality.

#### 3.1.2. Water Distribution

**Figure 3:**
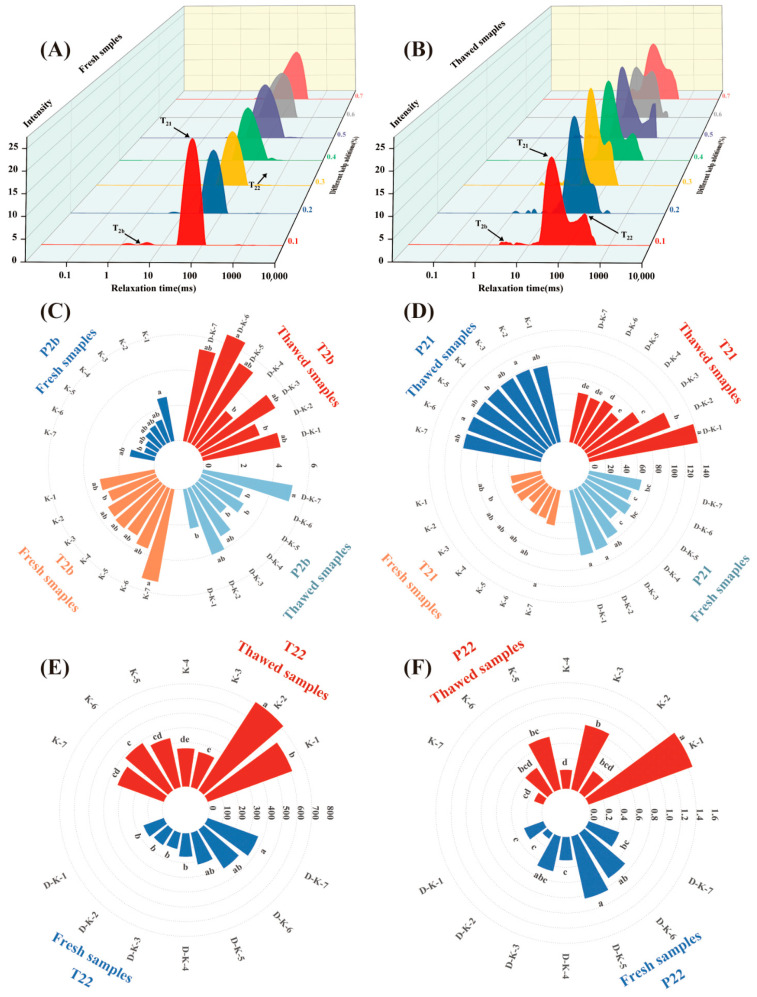
(**A**,**B**) represent the transverse relaxation time (*T2*) of seaweed–yak patties, prepared with varying seaweed addition levels, before and after freeze–thaw treatment. (**C**–**F**) illustrate the effects of different treatments on the relaxation proportions of *T2b*, *T21*, and *T22*, as well as on the (**C**) *T2* transverse relaxation time. (*T2b*: bound water; *T21*: immobilized water; *T22:* free water; *P2b*, *P21*, and *P22* correspond to the peak area ratios of water in each state). Lowercase letters in the figure denote differences among treatments at varying seaweed addition levels in both the fresh and freeze–thaw groups (*p* < 0.05).

Low-field nuclear magnetic resonance (LF-NMR) is an efficient method for analyzing water distribution and migration by measuring spin–spin relaxation time (*T2*) [37]. As shown in Figure 3A,B, the three-dimensional *T2* relaxation spectrum typically exhibits three characteristic peaks corresponding to different water states, as follows: *T2b* (0.1–1 ms) represents bound water, which is tightly associated with hydrophilic groups of muscle proteins and has low mobility but can exchange at the molecular level; *T21* (10–100 ms) represents immobilized water, accounting for approximately 85% of total muscle water and mainly located within the dense myofibrillar protein network, and its state is susceptible to external factors such as temperature and pressure, potentially migrating to the extracellular space; and *T22* (100–1000 ms) represents free water, located in the sarcoplasmic region, with high mobility and easy loss, serving as the main source of drip loss [38]. Water distribution significantly impacts the water-holding capacity and sensory quality of restructured yak meat patties. In Figure 3A, the restructured algae–yak patties before freeze–thaw contain virtually no free water. Figure 3B indicates that, as the seaweed addition level increases, *T2* relaxation times lengthen, suggesting that internal myofibrillar water migrates toward the extracellular space and the proportion of highly mobile water molecules increases [39]. Based on the three-dimensional *T2* spectra, Figure 3C–E illustrates relaxation time changes for the three water states before and after freeze–thaw at different seaweed addition levels. In Figure 3D, except for the 70%-addition group, the *T2b* values in all pre-freeze–thaw treatment groups show no significant change (*p* > 0.05); however, post-thaw *T2b* values exhibit an upward trend, indicating enhanced mobility of water molecules within the restructured yak steaks, likely due to weakened interactions between algal polysaccharides and proteins during thawing. The *T21* trends mirror those of *T2b*, as follows: in the 20%- and 40%-seaweed groups, *T21* increased from 40.93 ms and 29.67 ms (pre-freeze–thaw) to 109.96 ms and 57.94 ms (post-freeze–thaw), respectively, indicating a disruption of the myofibrillar protein network. The increase in *T22* values, reflecting expanded extracellular spaces, corresponds with changes in *T21*, suggesting that ice crystal expansion during thawing damages sarcolemma and myofibrils, promoting immobilized water migration. However, as the seaweed addition level increases, *T22* values gradually decrease; moreover, in particular, the 10%- and 20%-addition groups show significantly lower *T22* values than the other groups (*p* < 0.05), indicating that an appropriate level of seaweed can exert a water-retaining effect in meat products. As shown in Figure 3D–F, compared to fresh samples, *P21* values increase significantly with seaweed addition (*p* < 0.05), while *P22* values decrease, attributable to ice-crystal-induced compression damage to myofibrils during freeze–thaw. Notably, *P21* values in the 40%- and 60%-seaweed groups decrease by 42.49% and 41.35%, respectively, a significantly greater reduction than that observed in other groups, indicating that seaweed mitigates freeze–thaw damage by inhibiting increases in free water mobility and content. These results align with water-holding capacity (WHC) data, confirming the cryoprotective effect of seaweed. Nikoo et al. (2014) [40] reported that tetrapeptides extracted from fish skin gelatin can significantly inhibit the increase in bound water mobility and prevent its conversion to free water during repeated freeze–thaw cycles. A similar phenomenon was observed in pork patties supplemented with chitosan nanoparticles [41].

### 3.2. Physicochemical Properties of Reconstituted Seaweed–Yak Meat Patties

#### 3.2.1. pH and Color Analysis

As shown in Figure 2B, the pH of fresh alginate–yak patties ranged from 5.62 to 5.91. Moreover, as the alginate addition increased, the pH rose significantly (*p* < 0.05). This elevation is attributable to the intrinsic polysaccharide nature of alginate, consistent with studies on meat matrices containing dietary fiber [42]. Because seaweed is rich in negatively charged algal polysaccharides that interact with positively charged proteins to form aggregates, higher seaweed content in yak patties directly affects pH and thereby influences the water-holding capacity and textural properties [26]. This finding aligns with that of Alvarado and McKee (2007) [43], who concluded that elevated pH enhances water-holding capacity, which is directly related to meat batter texture. Compared to pre-freeze–thaw treatments, the pH of thawed restructured algae–yak patties decreased by an average of 0.06 ± 0.02. This reduction results from protein denaturation and proton release due to muscle tissue disruption during freezing, combined with increased solute concentration from drip loss during thawing, which lowers the sample pH [44].

Table 4 presents color parameters—lightness (L*), redness (a*), and yellowness (b*)—of restructured algae–yak patties before and after freeze–thaw at different seaweed addition levels. Both seaweed addition and freeze–thaw treatment had significant effects on color values (*p* < 0.05). With increasing seaweed addition, L* increased, with the 30% and 40% groups exhibiting the highest lightness. At each addition level, the thawed samples had higher L* than their pre-freeze–thaw counterparts. Since higher moisture content in meat products typically correlates with increased L*, the observed rise in L* may be due to the high water content of seaweed and its water-holding capacity during thawing [2].

Redness (a*) decreased significantly with increasing seaweed addition (*p* < 0.05), indicating a higher proportion of green pigments from seaweed. The thawed samples exhibited lower a* values than the pre-freeze–thaw samples, confirming that freezing induces myoglobin denaturation and affects color stability. However, the total color difference (Δ*E*) between pre- and post-freeze–thaw samples did not change significantly, consistent with Shan et al. (2009) [45], who reported minimal effects of spice and herb extracts on fresh pork color. This indicates that seaweed addition does not cause noticeable color changes during freeze–thaw, supporting its application in final products [26].

#### 3.2.2. Texture and Shear Force Analysis

The texture characteristics of restructured meat products—including hardness, springiness, cohesiveness, and adhesiveness—are core indicators for assessing product quality [4]. As shown in Table 5, increasing the proportion of seaweed addition led to a significant decrease in hardness, cohesiveness, and adhesiveness of the restructured algae–yak patties (*p* < 0.05), indicating that interactions between seaweed and yak meat markedly influenced TPA parameters. These findings are consistent with the shear force measurements (Figure 2D). Notably, springiness first increased and then decreased as the seaweed content rose, peaking at the 40% addition level, confirming that algal polysaccharides can form a stable network with yak meat. This phenomenon is primarily due to the excellent gelling ability and high water-holding capacity of algal polysaccharides, which aligns with the WHC results. These observations agree with those of Kim et al. (2018) [46], who reported that polysaccharide-containing meat products generally exhibit a softer texture, whereas adding sodium alginate and carrageenan to restructured duck ham increased hardness, cohesiveness, chewiness, and adhesiveness. Figure 2D further shows that the shear force values of thawed samples were generally higher than those of pre-freeze–thaw samples as seaweed addition increased, mirroring the trends in hardness. Importantly, the 30%- and 40%-seaweed groups showed the smallest increases in hardness—only 53.19% and 55.94%, respectively—validating previous conclusions that plant-based fiber additions enhance meat product hardness [2].

#### 3.2.3. Sensory Evaluation

A basic sensory evaluation covering odor, appearance, texture, and flavor was conducted on restructured algae–yak patties. Figure 2C summarizes sensory scores for off-odor, color, meat quality, and springiness at seaweed addition levels of 10–70%. The appearance scores ranged from 4.82 to 7.91, with the 20%- and 40%-addition groups scoring significantly higher than the others (*p* < 0.05), indicating that moderate seaweed incorporation enhances visual appeal. However, as seaweed content increased, color and odor scores progressively declined. Compared to previous studies, these results differ notably. Piñero et al. (2008) [47] reported that adding oat dietary fiber to conventional beef patties decreased sensory quality, with consumer acceptability scores approximately 23.5% lower than those of the control. Therefore, appropriate seaweed inclusion can not only improve the water-holding capacity of yak patties, but also enhance their overall sensory scores.

### 3.3. Changes in the Microstructure

#### 3.3.1. Magnetic Resonance Imaging (MRI)

**Figure 4:**
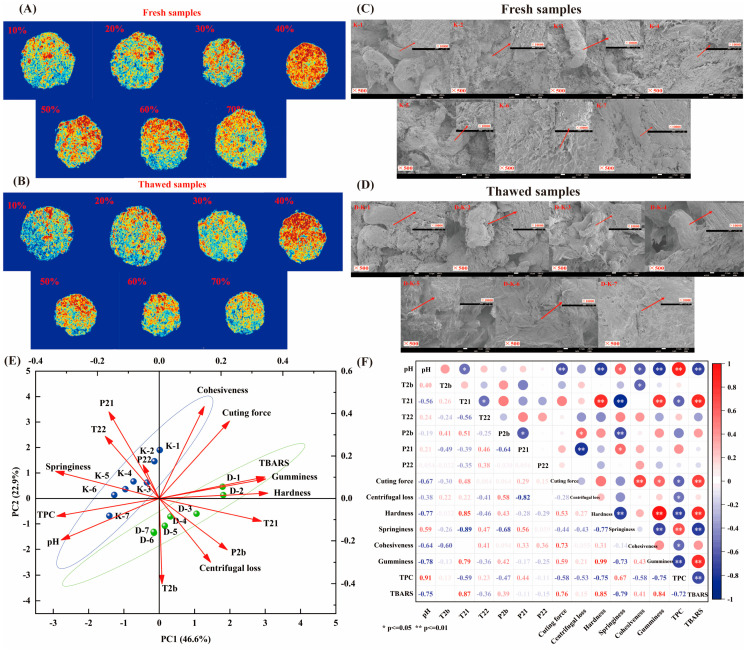
(**A**,**B**) display the MRI images of seaweed–yak patties under different treatments before and after freeze–thaw. (**C**,**D**) show the microstructure of the patties under the same conditions. (**E**,**F**) present the principal component analysis and correlation analysis among the various quality indicators, respectively.

MRI was used to quantitatively analyze the distribution of hydrogen protons in yak meat patties with varying levels of seaweed addition (in both fresh and thawed states) to elucidate the patterns of water migration and its spatial distribution characteristics. The MRI results (Figure 4A,B) show that red regions indicate areas of high hydrogen proton density (suggesting water enrichment), whereas blue regions reflect reduced proton signal intensity (indicating water loss), clearly demonstrating the differences in water status among the treatment groups. In the fresh samples, as the seaweed proportion increased, the area of the red regions gradually expanded. Notably, starting from the 40%-addition group, the proton signal was significantly enhanced, indicating a marked increase in water content. This phenomenon is consistent with the water-holding capacity results (Figure 2A). It may be attributed to the algal polysaccharides in seaweed absorbing water, after which the internal water is predominantly present as strongly bound water (Figure 3). The high hydrophilicity of algal polysaccharides facilitates their interaction with myofibrillar proteins, thereby enhancing the water-holding capacity of the yak meat patties. As shown in Figure 4B, compared with the thawed groups, the hydrogen proton intensity is considerably reduced; however, the 40%- and 50%-seaweed groups exhibit the smallest reduction in red region intensity, indicating that a moderate level of seaweed addition offers the best antifreeze effect for the patties. This observation aligns with our previous low-field nuclear magnetic resonance (LF-NMR) *T2* results (Figure 3), which demonstrated that the addition of a moderate amount of seaweed can restrict water migration during freeze–thaw cycles, leading to more tightly bound water molecules.

#### 3.3.2. Scanning Electron Microscopy (SEM)

SEM was employed to investigate the effects of varying seaweed addition levels on the microstructure of fresh and thawed yak meat patties. As shown in Figure 4C,D, with increasing seaweed content, the seaweed increasingly envelops the muscle fibers in a more uniform and thicker manner, and the sample surfaces become progressively denser and smoother (Figure 4C). This phenomenon is attributed to the water absorption and retention properties of bioactive substances such as algal polysaccharides present in seaweed. After absorbing water, these substances fill and compress the minced meat matrix, thereby rendering the samples more delicate and smooth. These findings are consistent with the previously observed water-holding capacity results (Figure 2A). Moreover, studies by Peng et al. (2023) [48] have reported similar conclusions, demonstrating that pork patties supplemented with non-hydrolyzed whey protein (NWP) and whey protein hydrolysate (WPH) exhibit a denser and smoother muscle fiber surface structure compared to those without such additives. The observations of the microstructure of thawed seaweed–yak meat patties revealed that samples with higher seaweed addition levels exhibited greater density post-thaw than those with lower levels. Notably, in the 40–70%- seaweed groups, observations at 10,000× magnification clearly showed that, after thawing, the muscle fiber surfaces were denser and smoother. In comparison to the 10–30% groups, the distribution of pores within the muscle fibers gradually decreased, and the fiber network structure became more uniform. These results suggest that the algal polysaccharides in seaweed can form a protective barrier, impeding the exchange of substances between the interior and exterior of the yak meat patties. Previous research has indicated that polysaccharides can interact with water molecules through hydrogen bonding, hydrophobic interactions, or electrostatic forces, thereby affecting the distribution and migration of water molecules around muscle proteins. This mechanism helps to reduce the damage incurred during the freezing and thawing processes [49].

### 3.4. The Effect of Different Seaweed Addition Levels on the Oxidation Characteristics of Reconstituted Steak

#### 3.4.1. MDA Content

Figure 2E illustrates the effect of varying seaweed addition levels on TBARS values in fresh and thawed yak meat patties. In fresh samples, malondialdehyde (MDA) concentrations ranged from 0.01 to 0.12 nmol/mg. As the proportion of *L. japonica* increased, MDA values decreased in a dose-dependent manner (*p* < 0.05), indicating enhanced inhibition of lipid oxidation at higher seaweed levels. However, in the 50%-, 60%-, and 70%-addition groups, MDA content rose slightly, likely due to a reduced meat-to-fat ratio limiting the substrate available for peroxidation. After freeze–thaw treatment, the MDA concentrations (0.07–0.35 nmol/mg) were universally elevated compared to those of the fresh patties. The thawed samples with 10–30% seaweed exhibited significant MDA increases relative to their fresh counterparts (*p* < 0.05). Notably, the 40%-seaweed group showed only a 9.76% rise in MDA post-thaw, a significantly smaller increase than that observed in the 10–30% groups, confirming that the higher phenolic content and DPPH radical-scavenging activity conferred by *L. japonica* effectively mitigate oxidative damage. These findings are consistent with those of Yim et al. (2019) [50], who reported significantly lower TBARS values in the longissimus muscle of Korean native black goats supplemented with 0.3% and 0.9% seaweed powder, demonstrating the in vivo antioxidative efficacy of seaweed bioactive substances. Another study reported that the addition of 0.5% (*w*/*w*) laminarin/fucoidan extract to cooked pork patties significantly reduced MDA accumulation during refrigerated storage (≤14 days), confirming the efficacy of seaweed-derived antioxidants in enhancing shelf life and oxidative stability within the food matrix [51].

#### 3.4.2. Total Phenolic Content (TPC)

Figure 2F shows the changes in TPC in fresh and thawed yak meat patties with varying levels of seaweed addition. Phenolic compounds exhibit polymorphic structural characteristics, with significant differences in molecular weight distribution that are closely linked to the intrinsic flavor formation of foods. The unique phenolic hydroxyl groups in these compounds provide antioxidant functionality through a conjugated aromatic ring system, with resonance stabilization effectively enhancing free radical scavenging efficiency [52]. In both the fresh and thawed groups, the TPC levels significantly increased with increasing seaweed addition (*p* < 0.05). However, when comparing fresh and thawed samples, the TPC levels in the thawed samples were lower than those in the fresh ones. Notably, at a 40% seaweed addition level, the TPC values in the fresh and thawed samples were 7.64 GAE/100 g and 5.32 GAE/100 g, respectively—a reduction of 30.42—which was significantly lower than the reductions observed at other addition levels. This indicates that a 40% seaweed addition is most beneficial for the antioxidant function of yak meat patties. This result is consistent with the MDA content measurements (Figure 2E).

#### 3.4.3. Investigation of DPPH Radical-Scavenging Activity

Due to its unique single-electron properties, DPPH is widely used as a standard probe molecule in in vitro antioxidant activity assays. This free radical reacts with hydrogen or electron donors, and its free radical scavenging efficiency is determined by measuring the attenuation of its electron spin resonance (ESR) signal intensity [30]. Figure 2G shows the DPPH radical scavenging ability of fresh and thawed yak meat patties at different levels of seaweed addition. Specifically, as the seaweed addition increased, the DPPH signal intensity in the yak meat patties significantly decreased, and the DPPH scavenging capacity of the thawed samples was reduced. Moreover, among the thawed samples, those with higher levels of seaweed addition exhibited a more pronounced decrease in DPPH signal intensity. These results are consistent with the MDA measurements (Figure 2E) and the total phenolic content measurements (Figure 2F).

#### 3.4.4. Non-Targeted Metabolomics

Based on the above antioxidant measurements, four groups of samples exhibiting the most pronounced differences were selected, namely the 10%- and 40%-seaweed-addition groups in both fresh and thawed states. To evaluate the clustering of metabolites in these four groups, a cluster analysis was performed on the significantly different metabolites, with each metabolite’s relative abundance represented by its peak area. The results are presented as a heatmap (Figure 2H). The heatmap clearly shows that the metabolites in the 10%- and 40%-seaweed yak meat patties differ significantly between the fresh and thawed states, with the fresh samples clustering into one group and the thawed samples into another; therefore, these two major clusters were subsequently aggregated into one overarching cluster. Among the metabolites, 4-(3-Hydroxy-2-naphthyl)-2-oxobut-3-enoic acid, cis-4-(1′-Hydroxynaphth-2′-yl)-2-oxobut-3-enoate, gamma-Glutamyl-gamma-aminobutyraldehyde, (5-L-Glutamyl)-L-glutamate, and 5-Oxoproline showed relatively higher abundance in the fresh samples of the 10%- and 40%-seaweed groups. In contrast, 4-Coumarate, Caffeic aldehyde, gamma-Glutamyl-gamma-aminobutyraldehyde, Isovalerylcarnitine (Car (5:0)), 2-Hydroxy-6-oxo-6-phenylhexa-2,4-dienoate, and (5-L-Glutamyl)-L-glutamate exhibited relatively higher abundance in the thawed samples of the 10% and 40% groups. Notably, (5-L-Glutamyl)-L-glutamate and Isovalerylcarnitine (Car (5:0)) displayed relatively high abundance across all four groups. It is particularly noteworthy that, although these two metabolites were present in all four sample groups, their relative abundances were highest in the thawed samples of the 10%-seaweed group, second highest in the thawed 40% group, and lowest in the fresh samples of the 40% group. These results corroborate the MDA, total phenolic content, and DPPH radical scavenging measurements, indicating that the addition of seaweed in yak meat patties not only reduces oxidation in meat products, but also significantly mitigates various oxidative processes during storage.

### 3.5. Correlation Analysis

To establish the correlations between the quality characteristics of fresh and thawed yak meat patties at different seaweed addition levels, a linear combination analysis was performed using principal component analysis (PCA) on color parameters, textural properties, water-holding capacity, water distribution, and oxidation indicators. As shown in Figure 4E, the PCA results revealed a total variance contribution of 66.9% (with PC1 accounting for 44.6% and PC2 for 22.9%), indicating a strong correlation between the quality characteristics of fresh and thawed patties across different seaweed levels. The PCA also showed that significant differences exist between the fresh and thawed groups, while the quality attributes of yak meat patties at different seaweed addition levels were highly similar. Wang et al. (2023) [53] investigated the effects of adding dietary fiber components to meat products on the physicochemical and sensory properties of restructured meat products, highlighting that the dietary fiber addition plays a critical role in determining quality. Our study further indicates that variations in seaweed addition levels not only affect the sensory attributes, but also influence product preservation.

Figure 4F displays the correlations among water distribution, physicochemical properties, textural quality, and oxidation characteristics in yak meat patties at various seaweed levels. This study found that *T21* was extremely positively correlated with hardness and MDA content; *P21* was significantly positively correlated with elasticity (*p* < 0.05) and extremely negatively correlated with centrifugation loss (*p* < 0.01); and pH value, elasticity, and total phenolic content were extremely negatively correlated with TBARS values (*p* < 0.01). These results indicate that incorporating an appropriate level of seaweed in yak meat patties not only enhances water-holding capacity, physicochemical properties, and sensory quality, but also reduces thawing loss and oxidative changes after frozen storage. These results corroborate the previous analysis.

### 3.6. Investigation of Yak Meat Protein Properties Before and After Freeze-Thaw Cycling

#### 3.6.1. Particle Size and Zeta Potential

**Figure 5:**
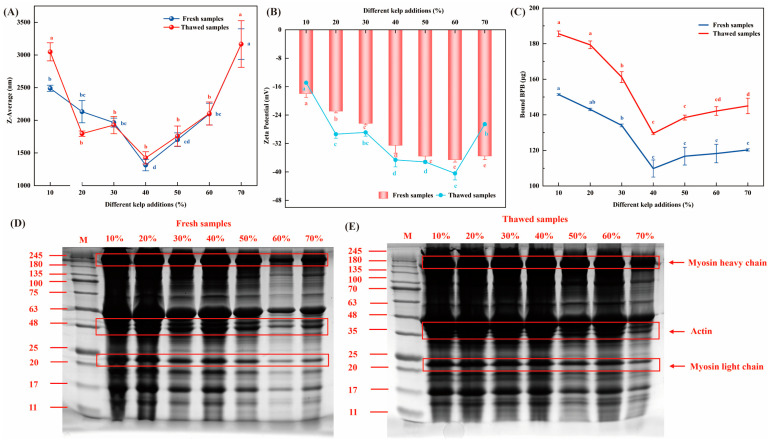
(**A**–**C**), respectively, represent the changes in particle size, zeta potential, and surface hydrophobicity at varying seaweed addition levels before and after freeze–thaw, while (**D**,**E**) display the SDS-PAGE profiles of myofibrillar proteins before and after freeze–thaw. Lowercase letters in the figure denote differences among treatments at varying seaweed addition levels in both the fresh and freeze–thaw groups (*p* < 0.05).

The aggregation state of MP can be reflected by changes in their particle size. During the thawing process, proteins may undergo oxidation-induced denaturation, leading to the exposure of internal hydrophobic regions, which in turn triggers intermolecular aggregation through hydrophobic interactions, thereby increasing the actual particle size [54]. Figure 5A shows that, with increasing seaweed addition, the particle size of yak MP initially decreases and then increases. In particular, the 40%-seaweed group exhibited a significantly lower MP particle size compared to that of the other groups (*p* < 0.05); however, in the 10%-seaweed group, the MP particle size of the thawed samples (3047.98 nm) was significantly higher than that of the fresh samples (2490.27 nm). This phenomenon is attributed to the formation and melting of ice crystals during freeze–thaw cycles, which disrupt the hydration layer on the protein surface, causing previously water-shielded protein groups to become exposed and interact, thereby triggering covalent cross-linking and aggregation between protein molecules [55,56]. In addition, freezing promotes the unfolding of MPs, and, due to hydrophobic interactions, this facilitates the formation of protein aggregates [56]. However, as the seaweed addition level increases, the particle size of MPs before and after freeze–thaw gradually decreases. For example, in the 40%-seaweed group, the particle size decreased by 336.22 nm, while, in the 40% and 50% groups, the particle size increased by only 109.17 nm and 55.01 nm, respectively. This indicates that seaweed exerts an antifreeze effect, preventing an increase in the average particle size of the meat emulsion, likely because the algal polysaccharides in seaweed can encapsulate the protein molecules, thereby inhibiting protein aggregation [57].

The stability of the system is directly related to the absolute value of the zeta potential, which is an important predictive indicator of the stability of colloidal dispersions. Figure 5B indicates that, with increasing seaweed addition, the absolute value of the zeta potential of yak MP significantly increases (*p* < 0.05). In the thawed samples with 40%, 50%, and 60% seaweed addition, the absolute zeta potential was the highest—being 59.31%, 59.93%, and 63.13% higher than that of the 10% group, respectively. This suggests that the polysaccharides in seaweed interact with the charges on the surface of the protein molecules, resulting in electrostatic repulsion that maintains system stability and prevents protein aggregation.

#### 3.6.2. Investigation of Surface Hydrophobicity

The hydrophobicity of the protein surface can serve as a key parameter for characterizing its conformational stability as it indicates both the exposure of hydrophobic groups during oxidation and the unfolding state of the protein molecules [54]. In this study, the bromophenol blue method was used for the quantitative analysis of surface hydrophobicity. As shown in Figure 5C, the hydrophobicity values for the groups with low algae addition (10%, 20%, and 30%) were 151.38 ± 0.42, 143.03 ± 0.68, and 134.06 ± 0.76 µg, respectively, which were significantly higher than that of the 40%-algae-addition group (109.82 ± 4.82 µg). According to the study by Chelh et al. (2006) [58], protein structures expose internal hydrophobic amino acids during unfolding and conformational changes, thereby increasing the surface hydrophobicity. This increase is mainly attributed to a higher protein content, which results in more exposed hydrophobic amino acids. A comparison of the samples before and after thawing indicated that the surface hydrophobicity of the proteins generally increased after freezing and thawing. Studies by Qiu et al. (2022) [59] and Zhang et al. (2023) [60] have also reported that frozen storage leads to increased surface hydrophobicity in proteins. These results confirm that severe protein denaturation due to frozen storage results in a significant reduction in hydration capacity. In contrast, the thawed samples in the 40%- and 50%-algae-addition groups displayed surface hydrophobicity values of 129.53 ± 0.64 µg and 138.47 ± 1.38 µg, respectively, corresponding to increases of 15.22% and 15.68% compared to those of the fresh group. The rate of increase in surface hydrophobicity was significantly lower in these groups than in the 10%- and 20%-algae-addition groups (18.41% and 20.25%, respectively). This is attributed to the addition of sodium alginate present in the algae, which acts as a cryoprotectant. The cryoprotectant minimizes the exposure of hydrophobic amino acids and prevents protein aggregation through intermolecular hydrophobic interactions, thereby stabilizing the secondary and tertiary structures of myofibrillar proteins [61]. Our results further explain the enhanced ability of proteins to bind with water molecules under the influence of an electric field.

#### 3.6.3. SDS-PAGE

Using SDS-PAGE techniques, we analyzed the structural changes in MPs in yak patties with different proportions of algae addition, both in fresh samples and after freeze–thaw treatment. As shown in Figure 5C,D, the myofibrillar protein bands correspond to the myosin heavy chain, actin, and myosin light chain. When comparing the fresh and freeze–thawed samples, there was no significant change in the distribution of the myosin heavy chain and actin. Notably, in the 40% and 50%-algae-addition groups, no new bands appeared, nor was there deepening of bands in the 15–25 kDa region after freeze–thaw treatment. This observation suggests that an appropriate amount of algae addition enables the algal polysaccharides not only to inhibit ice crystal growth, but also to encapsulate protein molecules within a glassy matrix, thereby protecting the proteins’ spatial structure and hydration state from damage, and consequently delaying protein degradation and oxidative cross-linking [61]. In contrast, the other sample groups exhibited varying degrees of band intensification at approximately 17 kDa, indicating that the addition of either too little or too much algae leads to muscle protein degradation during the freeze–thaw process. This degradation is likely due to the growth and recrystallization of extracellular ice during freezing and thawing, which may result in an increased concentration of solutes in the residual liquid phase and subsequent protein denaturation [62].

## 4. Conclusions

Adding seaweed as a dual-function, water-retention, and cryoprotective agent significantly enhances the quality of restructured yak patties subjected to freeze–thaw cycles. Incorporating 40% seaweed optimizes moisture distribution, preserves textural integrity, and enhances oxidative stability by forming a hydrogen-bonded polysaccharide–protein matrix. This fortification level creates a denser microstructure, maintains protein conformation, and increases phenolic antioxidant content, thereby effectively reducing thawing loss and lipid oxidation. Overall, appropriate seaweed inclusion offers a practical strategy for developing high-quality plant-based meat products with superior sensory and processing characteristics.

## Figures and Tables

**Table 1:** Sample information on seaweed–yak patties with varying levels of seaweed addition before and after freeze–thaw treatment.

IDX	SN	Sample Name	Details
1	K-1	Yak meat patties with 10% seaweed addition	5 g kelp + 45 g yak meat
2	K-2	Yak meat patties with 20% seaweed addition	10 g kelp + 40 g yak meat
3	K-3	Yak meat patties with 30% seaweed addition	15 g kelp + 35 g yak meat
4	K-4	Yak meat patties with 40% seaweed addition	20 g kelp + 30 g yak meat
5	K-5	Yak meat patties with 50% seaweed addition	25 g kelp + 25 g yak meat
6	K-6	Yak meat patties with 60% seaweed addition	30 g kelp + 20 g yak meat
7	K-7	Yak meat patties with 70% seaweed addition	35 g kelp + 15 g yak meat
8	D-K-1	Thawed yak patties with 10% seaweed addition	5 g kelp + 45 g yak meat
9	D-K-2	Thawed yak patties with 20% seaweed addition	10 g kelp + 40 g yak meat
10	D-K-3	Thawed yak patties with 30% seaweed addition	15 g kelp + 35 g yak meat
11	D-K-4	Thawed yak patties with 40% seaweed addition	20 g kelp + 30 g yak meat
12	D-K-5	Thawed yak patties with 50% seaweed addition	25 g kelp + 25 g yak meat
13	D-K-6	Thawed yak patties with 60% seaweed addition	30 g kelp + 20 g yak meat
14	D-K-7	Thawed yak patties with 70% seaweed addition	35 g kelp + 15 g yak meat

**Table 2:** Sensory evaluation criteria for the sensory properties of seaweed–meat patties.

Score	Odor	Color	Appearance Texture	Elasticity
9–10 (best)	Stronger intrinsic aroma; no unpleasant odor	Shiny surface	Tight; complete; clear texture	The most elastic; the depression disappears immediately after pressing
7–8 (better)	Inherent aroma; no unpleasant odor	More shiny	Tight; clear texture	More elastic; the depression disappears quickly after pressing
4–6 (good)	Light inherent aroma; slightly unpleasant odor	Slightly shiny	Not tight; not loose	More elastic; the depression disappears slowly after pressing
2–3 (general)	No inherent aroma; fishy odor	Slightly dull	Not tight; partially loose	Elastic; the depression disappears slowly after pressing
0–1 (poor)	Strong fishy odor	Matte	Not tight; loose	Inelastic; the depression does not disappear after pressing

**Table 3:** Effect of different seaweed addition ratios on thawing losses of reconstituted steaks.

Different Seaweed Addition Ratios (%)	10	20	30	40	50	60	70
Thawing loss (%)	8.2 ± 0.16 ^a^	6.47 ± 0.54 ^ab^	5.11 ± 0.19 ^bc^	3.69 ± 0.81 ^c^	4.31 ± 1.08 ^c^	4.94 ± 1.07 ^bc^	4.99 ± 1.29 ^bc^

Means followed by different letters within a column are significantly different at *p* < 0.05.

**Table 4:** Color parameters of restructured seaweed–yak meat patties with varying seaweed inclusion levels before and after freeze–thaw cycles.

Samples	L*	a*	b*	∆*E*
K-1	34.68 ± 1.51 ^abc^	8.75 ± 1.13 ^ab^	10.4 ± 1.01 ^abc^	3.58 ± 1.08 ^ab^
D-K-1	36.04 ± 1.64 ^ab^	10.49 ± 0.66 ^a^	11.13 ± 0.34 ^a^	
K-2	37.2 ± 0.89 ^a^	7.22 ± 2.33 ^bc^	10.9 ± 1.48 ^ab^	4.32 ± 1.74 ^ab^
D-K-2	34.21 ± 0.88 ^abc^	8.59 ± 0.86 ^ab^	11.04 ± 0.38 ^ab^	
K-3	36.23 ± 2.58 ^ab^	6 ± 1.95 ^c^	10.18 ± 1.5 ^abc^	3.1 ± 0.92 ^b^
D-K-3	36.24 ± 1.14 ^ab^	5.95 ± 0.33 ^c^	10.84 ± 0.26 ^ab^	
K-4	36.33 ± 4.35 ^ab^	5.24 ± 2.07 ^c^	10.5 ± 2.71 ^abc^	5.73 ± 3.88 ^ab^
D-K-4	32.8 ± 0.55 ^bc^	3.02 ± 0.34 ^d^	9.2 ± 0.32 ^bcd^	
K-5	28.82 ± 4.15 ^e^	3.13 ± 1.73 ^d^	8.3 ± 2.3 ^d^	4.45 ± 2.51 ^ab^
D-K-5	29.18 ± 1.15 ^de^	2.88 ± 0.51 ^d^	8.05 ± 0.4 ^d^	
K-6	31.94 ± 3.05 ^cd^	2.63 ± 1.35 ^de^	8.85 ± 1.54 ^cd^	3.55 ± 1.45 ^ab^
D-K-6	32.39 ± 0.91 ^c^	2.08 ± 0.25 ^de^	9.92 ± 0.16 ^abc^	
K-7	29.9 ± 7.62 ^de^	2.11 ± 1.55 ^e^	8.5 ± 3.04 ^d^	7.51 ± 4.81 ^a^
D-K-7	31.44 ± 0.78 ^cd^	0.71 ± 0.22 ^f^	8.92 ± 0.35 ^cd^	

Means followed by different letters within a column are significantly different at *p* < 0.05.

**Table 5:** Textural analysis of meat patties with different seaweed additions both fresh and after thawing.

Samples	Hardness/(N)	Springiness	Cohesiveness	Gumminess
K-1	816.04 ± 115.09 ^de^	0.27 ± 0.02 ^cde^	0.33 ± 0.004 ^a^	255.4 ± 37.39 ^de^
D-K-1	2320.11 ± 989.33 ^b^	0.16 ± 0.04 ^f^	0.25 ± 0.03 ^de^	560.94 ± 153.02 ^b^
K-2	1133.51 ± 65.06 ^cd^	0.34 ± 0.01 ^abc^	0.3 ± 0.01 ^b^	337.28 ± 10.2 ^cd^
D-K-2	3169.29 ± 567.48 ^a^	0.2 ± 0.01 ^ef^	0.27 ± 0.02 ^cd^	846.75 ± 182.41 ^a^
K-3	784.35 ± 85.16 ^de^	0.32 ± 0.05 ^abcd^	0.27 ± 0.02 ^cd^	209.59 ± 35.43 ^defg^
D-K-3	1675.75 ± 528.21 ^c^	0.23 ± 0.03 ^def^	0.25 ± 0.01 ^def^	413.94 ± 136.43 ^c^
K-4	497.72 ± 99.12 ^de^	0.4 ± 0.05 ^a^	0.26 ± 0.01 ^de^	127.8 ± 27.7 ^efgh^
D-K-4	1129.59 ± 300.45 ^cd^	0.25 ± 0.09 ^cde^	0.23 ± 0.02 ^efg^	256.2 ± 67.07 ^de^
K-5	434.44 ± 89.8 ^de^	0.35 ± 0.03 ^abc^	0.22 ± 0.01 ^fgh^	96.39 ± 20.24 ^gh^
D-K-5	1129.93 ± 229.52 ^cd^	0.27 ± 0.08 ^cde^	0.21 ± 0.01 ^gh^	240.52 ± 45 ^def^
K-6	417.7 ± 29.05 ^de^	0.38 ± 0.01 ^ab^	0.21 ± 0.01 ^ghi^	85.75 ± 7.76 ^gh^
D-K-6	1026.2 ± 177.31 ^cd^	0.28 ± 0.05 ^cde^	0.2 ± 0.01 ^hi^	202.77 ± 42.29 ^defg^
K-7	278.97 ± 10.37 ^e^	0.32 ± 0.005 ^abcd^	0.18 ± 0.01 ^i^	50.98 ± 3.98 ^h^
D-K-7	421.93 ± 54.78 ^de^	0.26 ± 0.04 ^cde^	0.23 ± 0.02 ^efg^	97.21 ± 12.98 ^gh^

Means followed by different letters within a column are significantly different at *p* < 0.05.

## Data Availability

The original contributions presented in the study are included in the article, further inquiries can be directed to the corresponding author.
